# Single versus dual orthogonal plating for comminuted midshaft clavicle fractures: a biomechanics study

**DOI:** 10.1186/s13018-020-01771-x

**Published:** 2020-07-09

**Authors:** Glenn N. Boyce, Andrew J. Philpott, David C. Ackland, Eugene T. Ek

**Affiliations:** 1 Melbourne Orthopaedic Group, 33 The Avenue, Windsor, Melbourne, VIC 3181 Australia; 2 Department of Orthopaedic Surgery, Bendigo Health, Bendigo, VIC Australia; 3 Department of Biomedical Engineering, University of Melbourne, Melbourne, VIC Australia; 4 Department of Surgery, Monash Medical Centre, Monash University, Melbourne, VIC Australia

**Keywords:** Clavicle, Fracture, Comminuted, Segmental, Biomechanical, Orthogonal, Internal fixation

## Abstract

**Background:**

Dual orthogonal plating of clavicle fractures may provide greater stiffness and strength than unilateral plate constructs and allow the use of lower-profile plates. We aim to biomechanically compare three clavicle plating constructs in a comminuted clavicle fracture model.

**Method:**

Fifteen clavicle sawbones were osteotomised, simulating a comminuted midshaft fracture and allocated to either: group 1, single superior plate (3.5 mm superior plate); group 2, combination plating (3.5 mm superior plate, 2.8 mm anterior plate) and group 3, dual mini-plates (two 2.8-mm orthogonal mini-plates). Specimens were biomechanically tested under torsion and cantilever bending. Construct stiffness (Nm/degree) and load to failure (Nm) were measured.

**Results:**

Group 2 had higher torsional (0.70 vs. 0.60 Nm/deg, *p* = 0.017) and cantilever bending stiffness (0.61 vs. 0.51 Nm/deg, *p* = 0.025) than group 1. Group 3 had lower cantilever bending stiffness (0.39 vs. 0.51 Nm/deg, *p* < 0.004) and load to failure (40.87 vs. 54.84 Nm, *p* < 0.01) than group 1. All dual plate constructs that catastrophically failed did so from fracture at the lateral ends of the plates. Single plate constructs failed due to plate bending.

**Conclusion:**

Dual orthogonal fixation with mini-plates demonstrated lower stiffness and strength than traditional superior plating. The addition of an anterior mini-plate to a traditional superior plating improved construct stiffness and may have a role in patients seeking early return to activity.

**Level of evidence:**

Basic science biomechanical study

## Introduction

Clavicle fractures account for 2.6% of all fractures, of which 80% occur in the midshaft [1]. Midshaft clavicle fractures have historically been managed largely nonoperatively. More recently, a greater appreciation of symptomatic malunion and higher rates of non-union than initially reported has driven a trend toward more frequent operative intervention [2–5]. The optimal fixation construct is still debated.

Plate osteosynthesis is widely used for stabilisation of midshaft clavicle fractures. A variety of plates and placement locations have been described. The ideal construct would provide adequate stability to facilitate union, permit early return to work and sport and minimise hardware bulkiness and the need for subsequent removal procedures. Metalware prominence is the most frequent cause for reoperation, required in 9–68% of patients, followed by construct failure, requiring revision in 0–16% of patients [6–12]. Plate bending, breakage and pull-out are recognised modes of plate failure [6–8]. Patients with comminuted injuries or those who return early to sport are at an increased risk [6–8, 13, 14].

The use of dual orthogonal plating may provide greater multidirectional stiffness and strength than traditional superior plating techniques [15]. The benefit of dual plating may be twofold: firstly, improved strength may reduce construct failure, and secondly, stronger constructs may allow the use of minimally prominent low-profile plates. Allis et al. recently compared dual mini-plates to standard superior plates for midshaft clavicle fractures and found significantly fewer reoperations for metalware irritation in the dual mini-plate cohort [16]. Few biomechanical studies exist comparing these constructs.

We aimed to compare the strength and stiffness of traditional superior plating against two dual orthogonal plating constructs in a comminuted midshaft clavicle fracture model. Our hypothesis was that dual orthogonal plating would provide greater stiffness and strength than traditional single superior plating.

## Materials and methods

### Specimen preparation

Fifteen left-sided adult clavicle sawbones of identical size were prepared based on previously reported biomechanical studies (Model 3308 Sawbones Worldwide, Vashon WA) [15]. The midportion of each clavicle was osteotomised using one standardised thermoplastic guide to remove a 90-degree butterfly fragment of 2-cm base, in order to represent inferior comminution or bone deficiency. Lack of bone to bone contact reduces the primary stability of the fracture and increases bending moments across the fixation construct.

Clavicle specimens were randomly allocated into three fixation groups that included the following:

- Group 1: single superior plate—Arthrex (Naples, FL, USA) 8-hole 3.5 mm (2.3 mm thickness) superior precontoured locking plate
- Group 2: combination plates—Arthrex (Naples, FL, USA) precontoured 8-hole 3.5 mm (2.3 mm thickness) locking plate positioned superiorly and Medartis (Basel, Switzerland) 10 hole 2.8 mm (1.6 mm thickness) olecranon locking plate positioned anteriorly
- Group 3: dual mini-plates—Medartis (Basel, Switzerland) 10-hole 2.8 mm (1.6 mm thickness) olecranon locking plates, one positioned superiorly and one positioned anteriorly

The two central holes of the precontoured 3.5-mm plate and the single central hole of the 2.8-mm mini-plates were left empty. All other screw holes were filled with bicortical locking screws. Thermoplastic guides were used to ensure plate positioning was replicated between specimens. Digital callipers were used to measure the plate position from the medial and lateral end of the clavicle. In all specimens, the position was consistent.

### Biomechanical testing

**Fig. 1:**
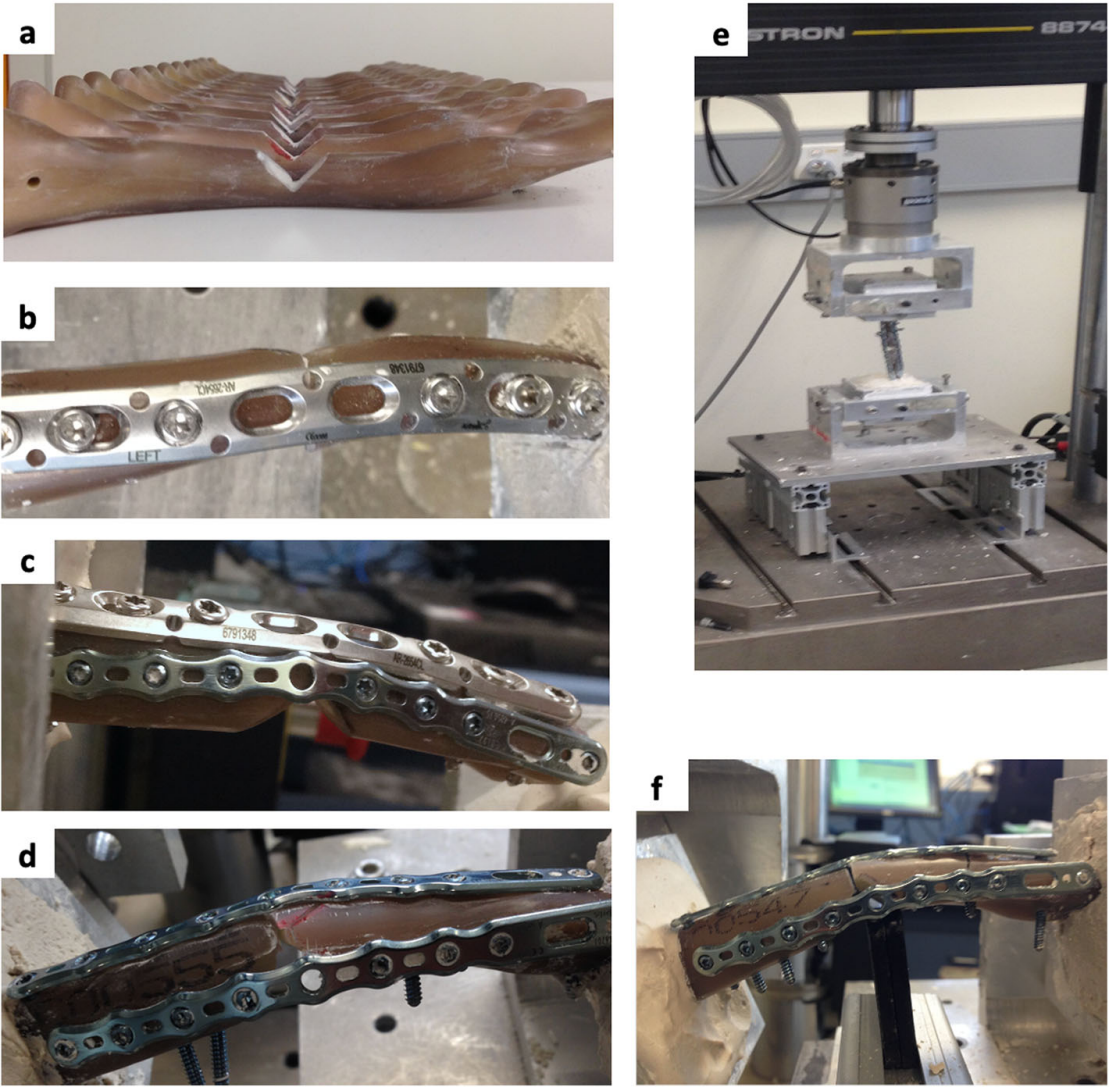
**a** Synthetic clavicle sawbones with 2 cm segmental osteotomy. **b** Group 1, single superior plate fixation. **c** Group 2, combination plate fixation. **d** Group 3, dual mini-plate fixation. **e** Biomechanical testing under torsional load. **f** Testing under cantilever bending load

Each clavicle specimen was embedded in customised potting fixtures using dental cement and mounted to an Instron Materials Testing Machine (Instron, Model 3521, Parker Hydraulics) [17, 18]. The sternal end of the clavicle was fixed to the lower crosshead of the Instron, while the acromial end was fixed to the upper crosshead (Fig. 1). The mechanical axis of the clavicle was positioned parallel with the vertical axis of the Instron.

The specimens were examined under torsion and cantilever bending [17]. Load rates were similar to those previously described [19]. Axial rotation was first tested by applying twisting motion about the longitudinal axis of the clavicle. An angular velocity of 0.5°/s was applied to the acromial end of the clavicle in the clockwise direction until 3° of rotation was achieved. The clavicle was then unloaded by 3° and a further loading of 3° in the counterclockwise direction was applied. Pilot data demonstrated a highly linear rotational displacement with increasing torque (*R*^2^ > 0.99) until the point of failure. The chosen range was used to prevent disruption to the construct, while ensuring sufficient data to define the linear region of the torque-angle curve. Stiffness during torsion (Nm/degree) was calculated from the gradient of the linear regression applied to each load-deformation curve. The *R*^2^ value was used as a measure of the goodness of the linear regression, and a minimum *R*^2^ of 0.95 was applied for all stiffness calculations.

Three-point cantilever bending was then performed on all constructs to failure. Each clavicle was positioned horizontally, with the proximal clavicle fixture rigidly fixed. A support was placed just medial to the fracture site, 95 mm from the distal end of the clavicle, in order to apply stress to the fracture construct rather than generating large moments at the proximal potting fixture [19]. The Instron crosshead applied a vertical downward force upon the acromial end of the clavicle, at a rate of 0.5 mm/s. Stiffness during cantilever bending (Nm/degree) was calculated from the gradient of the linear regression applied to each load-deformation curve. The *R*^2^ value was used as a measure of the goodness of the linear regression, and a minimum *R*^2^ of 0.95 was applied for all stiffness calculations. The bending moment (Nm) was calculated by multiplying the magnitude of downward force by the force moment arm (95 mm). Load to failure (Nm) was calculated as the bending moment which caused either 12° of construct flexure (approximately 20-mm vertical displacement) or the maximum bending moment prior to catastrophic failure of the construct resulting in loss of bending resistance.

### Statistical analysis

The Shapiro–Wilk test was used to assess data normality. A Levine’s test was then used to assess whether equal variances existed between groups. A 1-way analysis of variance (ANOVA) was then used to evaluate between-group differences, and Bonferroni post hoc testing was employed. Significance level was set as *p* < 0.05.

## Results

### Torsional stiffness

Under torsional loads, the combination plate construct (group 2) was significantly stiffer than the superior plate (group 1) (mean difference, 0.09 Nm/deg; 95% CI 0.01–0.17; *p* = 0.017) and dual mini-plate constructs (group 3) (mean difference, 0.15 Nm/deg; 95% CI 0.07–0.23; *p* < 0.001) (Table 1). The superior plate was not significantly stiffer than the dual mini-plate construct (mean difference, 0.05 Nm/deg; 95% CI 0.03–0.13; *p* = 0.291).

**Table 1:** Mean torsional stiffness, cantilever bending stiffness and load to failure of the fixation constructs

	Torsional stiffness (Nm/degree)	Cantilever bending stiffness (Nm/degree)	Cantilever bending load to failure (Nm)
Single superior plate	0.60 (± 0.08)	0.51 (± 0.01)	54.84 (± 5.18)
Combination plate	0.70 (± 0.06)	0.61 (± 0.05)	60.78 (± 7.41)
Dual mini-plate	0.55 (± 0.07)	0.39 (± 0.06)	40.87 (± 3.43)

### Cantilever bending stiffness

**Fig. 2:**
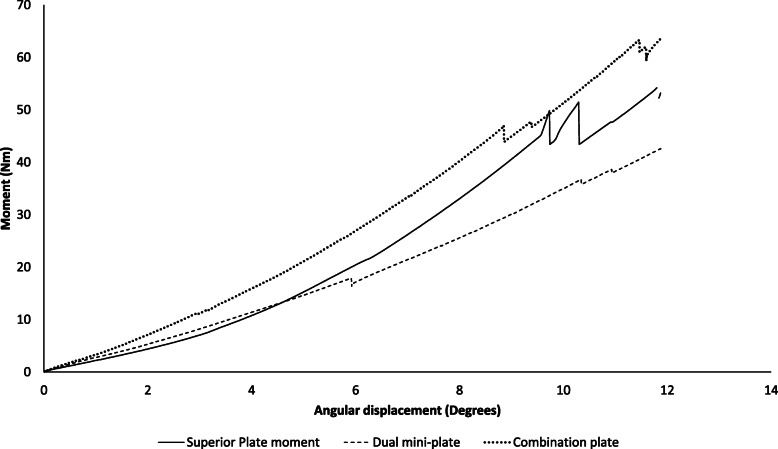
Load-displacement curve displaying mean angular displacement occurring with each fixation construct under cantilever bending force

Under cantilever bending loads, the combination plate construct was significantly stiffer than superior plate (mean difference, 0.09 Nm/deg; 95% CI 0.01–0.18; *p* = 0.025) and dual mini-plate constructs (mean difference, 0.22 Nm/deg; 95% CI 0.13–0.30; *p* < 0.001). The superior plate was significantly stiffer than dual mini-plate construct (mean difference, 0.12; 95% CI 0.04–0.21; *p* = 0.004) (Fig. 2).

### Cantilever bending load to failure

Under cantilever bending loads, the combination plate construct had significantly higher load to failure than dual mini-plate construct (mean difference, 19.91; 95% CI 10.10–29.72; *p* < 0.001), but not the superior plate construct (mean difference, 5.94; 95% CI 3.87–15.76; *p* = 0.354). The superior plate had significantly higher load to failure than the dual mini-plate construct (mean difference, 13.97; 95% CI 4.16–23.78; *p* = 0.006).

### Failures

Five construct failures occurred before 12° of displacement: in the superior plate group, two constructs failed through plate bending at the fracture site, in the combination plate group, two constructs failed through clavicle fracture at the most lateral screw hole and in the dual mini-plate group, one construct failed through clavicle fracture at the most lateral screw hole.

## Discussion

This study compared two feasible orthogonal plating alternatives to traditional superior plate fixation in a comminuted midshaft clavicle fracture model. The addition of an anterior mini-plate to traditional superior plate fixation increased construct stiffness. In contrast, fixation with dual orthogonal mini-plates provided the lowest stiffness and strength, and our findings would caution its routine use.

Present challenges in clavicle fixation are implant prominence and fixation failure. An optimum construct would be minimally prominent, have adequate strength to resists the loads encountered and provide appropriate stiffness to facilitate fracture union. Construct failure has been reported through plate bending or pull-out, when the fracture is subject to excessive forces prior to union [6–8]. Risk factors for construct failure are comminuted fracture patterns [14], the use of plates as bridging constructs [13] and early return to sport [20]. One study reviewing professional athletes who returned to contact sport within 6 weeks of surgery reported a 33% incidence of plate bending [20]. Dual orthogonal plating may reduce the incidence of construct failure and permit the use of low-profile plates to reduce metalware irritation.

Allis et al. reviewed 44 patients with midshaft clavicle fractures managed with either 3.5-mm superior plating or dual mini-fragment plate fixation [16]. Reoperation rates were significantly lower in patients managed with dual mini-fragment plates than those managed with a 3.5-mm superior plate, primarily due to less frequent metalware irritation (0% vs. 29%, *p* = 0.008). All patients achieved union and no significant difference in functional outcomes was observed. The authors concluded that dual mini-fragment plate fixation is a viable treatment that may reduce reoperation with no compromise to clinical outcomes.

Czajka et al. reviewed 81 patients with midshaft clavicle facture managed with dual mini-fragment plate fixation [21]. Three patients (4%) required secondary surgery for soft tissue irritation, 2 (2%) for infection and 1 (1%) for mechanical failure. The authors concluded that dual mini-fragment plating resulted in excellent functional outcomes and low rates of symptomatic metalware, albeit at higher implant cost.

Few biomechanical studies exist comparing unilateral to orthogonal plating in clavicle fractures. Prasarn et al. undertook a clinical and biomechanical analysis of transverse clavicle fractures managed with dual mini-plates [15]. Dual mini-plate fixation with a superior 2.7-mm plate and anteroinferior 2.4-mm plate were compared to traditional unilateral 3.5-mm plate fixation in either a superior or anteroinferior position. Tested on sawbones, dual mini-plates had comparable stiffness to unilateral plating under axial and rotational loads. Under superior-inferior and anterior-posterior four-point bending loads, dual plating was more rigid than unilateral plating when the unilateral plate was stressed across its broad edge, but weaker than a unilateral plate stressed across its narrow edge. Seventeen patients were managed using the technique in a clinical setting and all achieved union, with no metalware removal procedures required. The authors concluded that dual mini-plating provided better multidirectional stability than single plate fixation, with excellent clinical outcomes and reduced incidence of metalware prominence requiring a second procedure.

Ziegler et al. compared dual mini-fragment plating to traditional unilateral plating in cadaver clavicles with a simulated inferior butterfly fragment [22]. No significant differences in stiffness or strength were observed between dual mini-fragment plating compared to traditional unilateral plating. The authors concluded that dual small-plate fixation is a viable option for midshaft clavicle fractures. This has been further supported by a recent finite element analysis study by Zhang et al. who demonstrated similar results [23].

The present study examined a sawbone clavicle fracture model with inferior comminution. We compared three constructs: traditional 3.5-mm superior plating, traditional 3.5-mm superior plating with an additional anterior mini-plate and dual mini-plating. Unilateral superior plating was chosen as a control because it is a widely utilised means of fixation and has been found biomechanically preferable to anterior plating in some studies [19, 24, 25]. Under cantilever bending, traditional superior plate fixation failed at 55 Nm (577 N at 95 mm). This is favourable to load to failure reported in prior studies: Celestre et al. reported failure at 36 Nm (300 N at 120 mm) with 3.5-mm superior locking plates [19], Ziegler et al. reported failure at 20 Nm (201 N at 100 mm) with a 3.5-mm superior plate [22], Smith et al. reported failure at 18 Nm with a superior precontoured plate [26] and Drosdowech et al. reported failure through plate bending at 8 to 22 Nm in a model with an inferior cortical defect [27]. We suspect differences in fixation constructs, clavicle models and testing methodology are likely the source of the substantial interstudy variation observed.

We then assessed the impact of adding an additional 2.8-mm anterior mini-plate. The effect was to significantly increase construct stiffness without increasing load to failure. Finally, we tested dual mini-plating. Dual mini-plating provided the lowest stiffness and strength of all constructs. Similar to Zeigler et al.’s results, we found orthogonal plating constructs to fail due to clavicle fracture propagating from the final screw hole, in contrast to unilateral plating, which failed through plate bending. If orthogonal plating is to be used, we recommend staggering the plates to minimise the stress riser created.

It is unclear how strong clavicle fixation constructs need to be. Measuring the forces acting on the clavicle in vivo is challenging and therefore studies have relied on cadaveric testing and computational modelling. Taylor et al. used a computerised model and found the maximum forces experienced by the clavicle midshaft during eating were in a superior to inferior direction [28]. Hoogervorst et al. used the Delft shoulder and elbow model to estimate the load transmitted through the clavicle during activities of daily living [29]. The maximal force experienced was 97 N, was compressive in nature and occurred during shoulder abduction. The model did not account for any items in the subject’s hand or force applied through the hand during activity. Iannotti et al. assessed midshaft clavicle forces using a load cell mounted to 6 cadaveric clavicles and similarly found the greatest force to be an axial compressive force, occurring during shoulder abduction [24]. We surmise intermittent forces of far greater magnitude are experienced by patients during postoperative rehabilitation, particularly those who return early to sport or manual work.

The present study has a number of limitations. Firstly, this study used composite clavicle models that do not model the inhomogeneous anisotropic material properties of cortical and trabecular bone; however, synthetic clavicle models have been widely used in previous studies, closely replicate the properties of cadaver bone and minimise inter-specimen variability [19, 25, 30–32]. Failure of orthogonal plating constructs occurred through fracture of the composite bone at the final screw hole and it is unclear if this would be the case in a clavicle in vivo. Secondly, loading of clavicles was performed under unidirectional loading conditions. This provides interpretable results and permits interstudy comparison, however, may underestimate the multidirectional forces and ligamentous and muscular supports acting on the clavicle in vivo. Thirdly, cyclic loading and long-term integrity were not evaluated and therefore these findings reflect the immediate postoperative construct performance only. Finally, the clavicle fracture model tested had an inferior wedge defect, which represents ‘worst case scenario’ inferior comminution. In practice, reduction of inferior bony fragments or grafting to the defect would alter the biomechanical properties of fixation constructs.

## Conclusion

The strength of the present study is that it compares two feasible orthogonal plating alternatives to traditional superior plate fixation in a comminuted midshaft clavicle fracture model. Our results do not support the routine use of dual mini-plates due to their lower stiffness and strength than traditional superior plate fixation. The addition of an anterior mini-plate to traditional superior plate fixation increased construct stiffness and may have value in patients seeking early return to activity.

## Data Availability

The datasets used and/or analysed during the current study are available from the corresponding author on reasonable request.

## Abbreviations

Nm: Newton metres
Deg: Degrees
